# Enhanced Reflection of GaAs Nanowire Laser Using Short-Period, Symmetric Double Metal Grating Reflectors

**DOI:** 10.3390/nano12091482

**Published:** 2022-04-27

**Authors:** Qun Yu, Wei Wei, Xin Yan, Xia Zhang

**Affiliations:** 1 School of Electronics and Communication Engineering, Guangzhou University, Guangzhou 510006, China; yuqun@gzhu.edu.cn; 2 State Key Laboratory of Information Photonics and Optical Communications, Beijing University of Posts and Telecommunications, Beijing 100876, China; xyan@bupt.edu.cn (X.Y.); xzhang@bupt.edu.cn (X.Z.)

**Keywords:** nanowire, metal grating, nanolaser

## Abstract

Owing to the high contrast of the refractive indices at the end facets of a nanowire, lasing emission can be achieved in an individual nanowire without external, reflected feedback. However, the reflection provided by the end facet is not high enough to lower the threshold gain, especially for nanowires with smaller diameters. This work proposes a novel structure of nanowire laser partially sandwiched in double Ag gratings. Compared to a nanowire with a single metal grating or without a grating, the parallel double metal gratings play the reflector role with higher reflectivity to enhance the round-trip feedback and reduce the threshold gain. The reflective properties are calculated using the finite elements method. Simulation results show that a high reflectivity of more than 90% can be achieved when the number of periods is more than 8. The reflectivity of double gratings is 2.4 times larger than that of the nanowire end facet for large nanowire diameters. When the nanowire has a small diameter of 150 nm, the reflectivity of double gratings is 17 times larger than that of the nanowire end facet. Compared to a single grating, the reflective performance of double gratings is much better. Owing to the highly reflective properties of the double gratings, nanowires partially sandwiched in the double gratings can realize lasing emission at a very low threshold gain, and the period of the grating can be very short to benefit on-chip interconnection systems.

## 1. Introduction

Since the first laser realized by Maiman fifty years ago, and with the development of semiconductor techniques, laser devices are moving towards miniaturization and integration. Nanolasers, which generate coherent light at nanoscale, have attracted significant interest and have demonstrated fantastic applications in sensing, spectroscopy, optical computing and optical interconnects [1,2,3,4,5,6]. Technical progress in semiconductor epitaxial growth and the fabrication process is driving the development of nanolasers [7,8,9,10,11,12]. Semiconductor nanowires have wire-like cylindrical geometry and strong two-dimensional confinement of electrons, hole and photons. The simultaneous provision of both gain medium and an optical cavity for lasing emission makes them one of the ideal candidates for nanolasers [13,14,15,16]. Up-to-date, room-temperature lasing emission covering the optical spectrum from ultraviolet to near-infrared was realized in ZnO, GaN, CdS, and even GaAs nanowires [17,18,19,20,21]. Among them, a GaAs nanowire is considered to be a prime candidate for advanced opto-electronic devices due to its direct band gap and high electron mobility [22]. However, the end facet of a GaAs nanowire offers weak reflection for the round-trip light propagation inside the nanowire when its tiny diameter is compared with the near-infrared emission wavelength [23], especially for smaller diameters approaching the diffraction limit. Thus, a longer nanowire is required to compensate for the threshold loss induced by the low end-facet reflection and to provide greater round-trip amplification. However, the overlong nanowire axial dimension compared to the radial dimension impedes potential applications in high-density photonic integration.

Thus, in this paper, a novel structure consisting of a nanowire partially sandwiched in double Ag gratings is proposed to provide end reflection for the nanowire. The double short-period gratings are employed to be highly reflective mirrors to reduce the threshold gain and shorten the nanowire length, while the other end-facet reflection is still provided by the Au cap. The Au cap, having an extremely small size, provides a strong reflection for the light-wave guided inside the nanowire when the nanowire diameter is large. The double gratings and the Au cap, together with the semiconductor nanowire, form the optical resonant cavity. However, for smaller nanowire diameters, the reflection provided by the Au cap becomes weaker. Thus, the end part with the Au cap of the nanowire can be symmetrically sandwiched in the double metal gratings to provide high reflection by the metal gratings rather than the Au cap to further reduce threshold gain. To reveal the mechanism of the strong reflection of the double gratings, the finite elements method (FEM) is employed to perform numerical simulation of propagation and reflection properties. This work is a piece of extended research based on our previous work on enhanced reflection of single gratings [24]. Furthermore, by comparing the reflection and cavity properties of double gratings with the single grating, the short-period double gratings with strong reflection can effectively reduce the threshold gain and axial length of the nanowire.

## 2. Sandwich Structure of a Nanowire and Double Gratings

**Figure 1:**
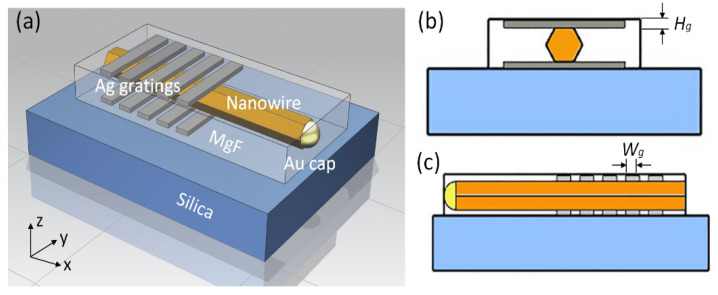
Schematic diagrams of the nanowire partially sandwiched in the double Ag gratings. (**a**) 3D structure, (**b**) Y-Z cross-section, (**c**), X-Z cross-section.

The proposed novel structure is a sandwich structure that consists of a GaAs nanowire inside the double Ag gratings, the schematic diagram of which is shown in Figure 1. On the silica substrate is the sandwich structure enclosed by the low-refractive-index material of MgF. The MgF material is adopted here to fix and fill the sandwich structure and further form the refractive index difference. The double Ag gratings are symmetrically placed on the upside and downside surfaces of the nanowire. The Ag permittivity is described by the Drude–Lorentz model:

(1) $$\varepsilon \left(\omega \right)=1+\sum_{k}\frac{\Delta \varepsilon_{k}}{-\omega^{2}-a_{k}\left(i\omega \right)+b_{k}}$$

where $\Delta \varepsilon_{k}$, $a_{k}$ and $b_{k}$ are constants that provide the best fit for silver when compared with the optical constant data of silver given by Palik et al. [25]. It is an extension of the Drude model incorporating additional Lorentz terms to better describe the transported properties of electrons in metals [26,27,28]. The terms of $a_{k}$ and $b_{k}$, respectively, denote the damping frequency of the electron gas and the effective electron collision frequency. To investigate the impact of grating parameters on the reflective properties, we use *Λ* to denote the grating period and $W_{g}$ and $H_{g}$ to denote the width and height of the grating teeth. The duty cycle of the grating is fixed at 50%. The double gratings have the same parameters and both are placed at the end part of the GaAs nanowire with dielectric interface to make the round-trip light-wave inside the nanowire interact with the gratings and strengthen the end reflection. The other part of the nanowire is only enclosed by MgF without gratings. The Au cap on its top is a gold nanoparticle used as catalyst during the epitaxial growth of the GaAs nanowire [20,21]. To get close to the experimental conditions, we assume the existence of the Au cap in the simulation. Its size is approximately equal to the diameter of the nanowire. Due to the strong reflective capability of the metal, the Au cap is naturally a highly reflective mirror with reflectivity more than 70% dependent on the nanowire diameter, while the reflective capability of the other end-facet dielectric interface is much weaker, especially for the nanowire with a diameter of less than 200 nm. To overcome the mirror loss induced by the dielectric end facet, the nanowire length needs to be long enough to satisfy the threshold gain. Thus, double gratings only need to be placed at this end part to enhance the reflection and compensate for the mirror loss. Consequently, the Au cap and double Ag gratings act as reflectors to form an optical resonant cavity.

## 3. Results

**Figure 2:**
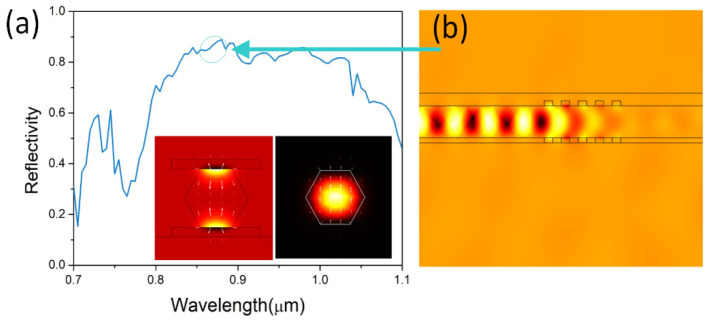
Reflective properties of double gratings. (**a**) Reflectivity spectrum of the nanowire with double gratings. (**b**) Profile of *H_z_* of the propagating mode. Insets are electromagnetic energy density profiles of mode *HE*_11*a*_ for the nanowire sandwiched in the silver grating teeth and in the air. The diameter of the nanowire is 250 nm.

The properties of mode, propagation and reflection of the double gratings are numerically calculated using FEM and are shown in Figure 2. Without the double gratings, the waveguiding mode *HE*_11*a*_ is centrally confined in the nanowire. In the presence of the double gratings, the mode interacts with the metal gratings and forms plasmonic modes together with a refractive index difference. The periodic refractive index difference of waveguiding mode *HE*_11*a*_ for the nanowire in MgF and thr symmetric grating teeth, respectively, result in the reflection of the light-wave by the double gratings. The double gratings provide a wide reflective spectrum for the light-wave inside the nanowire. By fixing the grating period and teeth height at 140 nm and 40 nm, the reflective spectrum covers the gain spectrum of the GaAs nanowire. Owing to the high refractive index contrast, the short-period double gratings with a period number of 5 can achieve a high reflectivity of higher than 85%. As shown in the standing wave depicted in Figure 2b, the high reflection provides strong feedback for the cavity.

**Figure 3:**
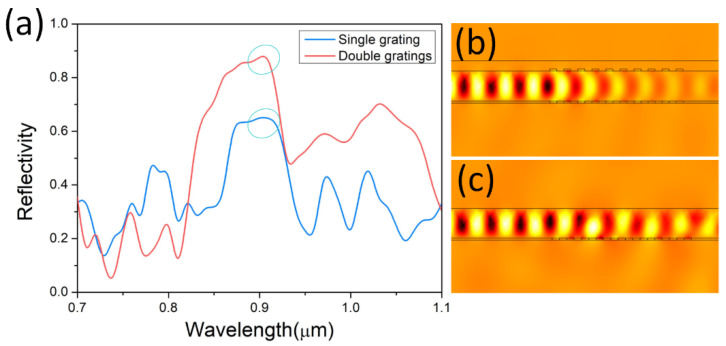
Reflective properties of single grating and double gratings. (**a**) Reflectivity spectra of the nanowire with single grating (blue) and double gratings (red). Profiles of *H_z_* of the propagating mode for the nanowire sandwiched in double gratings (**b**) and placed on single grating (**c**). Insets are modal profiles of *HE_11a_* for the nanowire sandwiched in double gratings and in air, respectively.

With similar refractive index contrast, a single grating can also provide high reflection and act as a reflector, which was elaborated on in our previous work [24]. To compare the reflective properties between double gratings and a single grating, reflective spectra are numerically calculated and demonstrated in Figure 3. With the same grating period, teeth height and number of the grating period, the reflection of double gratings is much stronger than that of a single grating. The reflectivity of the double gratings is as high as 88.7%, while the reflectivity of the single grating is only 65.3%. As in the profiles of *H_z_* of the propagating mode shown in Figure 3b,c, the waveguiding mode interacts more strongly with the double gratings than the single grating. Most of electromagnetic energy is reflected by the double gratings, rather than leaking out of the nanowire.

**Figure 4:**
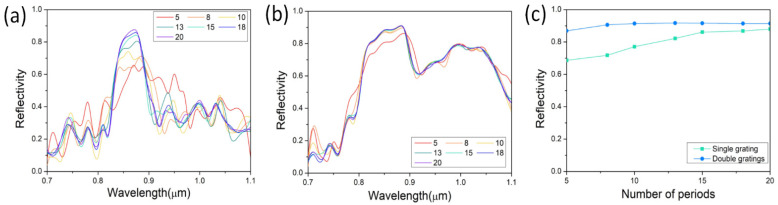
Reflective properties of gratings dependent on number of periods. Reflective spectra of various number of periods for nanowire (**a**) on single grating and (**b**) sandwiched in double gratings. (**c**) Comparison of reflectivity between nanowire on single grating and sandwiched in double gratings.

Reflective properties, including bandwidth and the central wavelength of the reflection spectrum, are dependent on the structural parameters of the grating. The reflection intensity is directly decided by the number of the grating periods. The reflectivity of single grating and double gratings as functions of the number of the grating periods are depicted in Figure 4. The grating pitch and teeth height are assumed to be 140 nm and 20 nm, respectively. The reflectivities of a single grating and double gratings both become larger as the number of the grating period increases. However, the reflection of the single grating has a stronger relation to the number of the grating period than that of the double gratings. When the number of the grating period increases from 5 to 15, the reflectivity of the single grating increases fast. The reflectivity changes slowly when the number of the grating period continuously increases and reaches its maximum at number of 20, while, for the reflectivity of the double gratings, it changes much more mildly with the increasing number of the grating period. Only for the increment from 5 to 8 does the reflectivity increase a lot. After that, the reflectivity only changes a little and remains at a high level. As shown in Figure 4c, the double gratings have a stronger reflection than the single grating no matter how much the grating period varies. When the number of the grating period is high enough, the reflectivity difference becomes smaller, and the single grating has a similar reflection to the double gratings. For the reflectivity difference with a small number of the grating period, the reflection of the double gratings is still much stronger. The double gratings only need 5 periods of grating to achieve similar reflection intensity, compared to the single grating, which requires 20 periods, which effectively shortens the length of the nanowire laser. Thus, the shortest gratings with high reflectivity can only be 700 nm in length. To obtain higher reflectivity, the gratings are also very short at 1400 nm. In the following calculations, all the numbers of the grating period are assumed to be 10.

**Figure 5:**
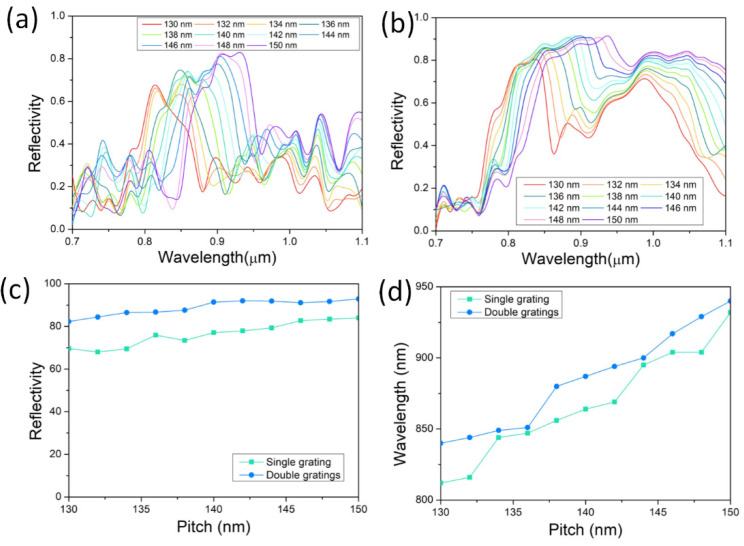
Reflective properties of gratings dependent on pitch. Reflective spectra of various pitch for nanowire (**a**) on single grating and (**b**) sandwiched in double gratings. Comparison of (**c**) reflectivity and (**d**) central reflective wavelength between nanowire on single grating and sandwiched in double gratings.

As the duty cycle of the grating is fixed at 50%, the grating pitch *Λ* is crucial to the central wavelength of the reflective spectrum. The reflective spectra and central wavelength of the single grating and double gratings for various *Λ* values are shown in Figure 5. Both the central wavelengths of the reflective spectra for the single grating and the double gratings experience a red-shift as *Λ* increases from 130 to 150 nm. The central wavelength of the reflective spectra for the double gratings experiences similar increases to that for the single grating with the increasing *Λ*. Aside from this, the reflections of both the double gratings, and the single grating gently become stronger as *Λ* increases. The reflection of the double gratings still performs more strongly than that of the single grating for all the pitches. Thus, through optimizing the pitch *Λ*, the reflective spectrum of the double gratings can be adjusted within or covering the gain spectrum of the GaAs nanowire, mostly ranging from 850 to 880 nm.

**Figure 6:**
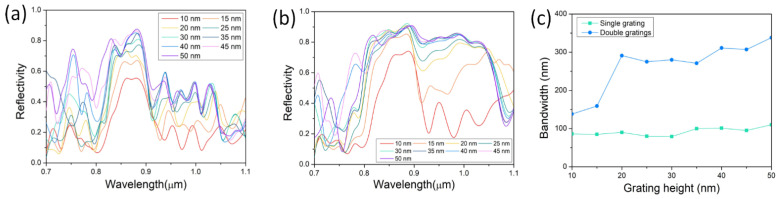
Reflective properties of gratings dependent on height of grating teeth. Reflective spectra of various height of grating teeth for nanowire (**a**) on single grating and (**b**) sandwiched in double gratings. (**c**) Comparison of reflectivity between nanowire on single grating and sandwiched in double gratings.

Similar to the number of the grating period, the height of the grating teeth also impacts on the reflection intensity of the grating. As depicted in Figure 6, both the reflectivity of the double gratings and the single grating increases with the increase of teeth height from 10 to 50 nm. They both have the smallest reflectivity at a teeth height of 10 nm. For the single grating, the reflectivity grows faster with increasing teeth height, while, for the double gratings, the reflectivity changes little for all teeth heights except for 10 and 15 nm. When the teeth height is small, the interaction between the guiding mode and the metal grating is weak. The weaker interaction with the decreasing teeth height results in the weaker reflection. In addition, the bandwidth of the reflective spectrum for the double gratings increases with the increasing height of grating teeth, covering more gain spectra of different compositions of compound semiconductor materials. The bandwidth is defined as the FWHM, i.e., full width at half maximum. Due to the disturbance of multiple peaks, the bandwidth can only be estimated to demonstrate the varying trend. It is necessary to note that simulation results may not be very precise at a teeth height of 10 nm for the limitation of the classic Drude model and its extensions of the adopted Drude–Lorentz model at about 10 nm [29]. The reflective spectrum for teeth height of 10 nm here is only for demonstrating the varying trend and making comparisons.

**Figure 7:**
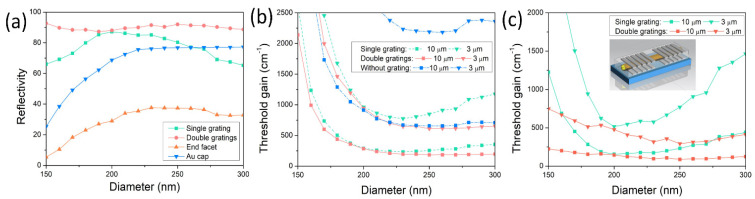
Reflective and threshold gain properties for different nanowire diameters. (**a**) Reflective spectra as functions of nanowire diameter for nanowire on single grating, in sandwiched double gratings, bare nanowire end facet and Au cap. (**b**) Threshold gain as functions of nanowire diameter for nanowire with length of 3 and 10 μm on single grating, sandwiched in double gratings and without grating. (**c**) Threshold gain as functions of nanowire diameter for nanowire with length of 3 and 10 μm on symmetric single gratings and symmetrically double gratings. The number of grating periods is 10.

The development of photonic integration towards high density requires components with reducing dimensions. However, the end-facet reflectivity of the nanowire dramatically decreases with the decreasing diameter, which is shown in Figure 7. Compared to the reflectivities of the Au cap, single grating and double gratings, the end-facet reflectivity is quite low, and the end facet even cannot provide enough reflection for the waveguiding mode at a diameter of around 150 nm, while the reflectivity of the double gratings remains at around 90% for all the nanowire diameters and a high level even for small nanowire diameters. Thus, the metal double gratings are much more significant for enhancing reflection and reducing threshold gain for nanowires with small diameters. Furthermore, the reflectivity of the double gratings is higher than that of the single grating for all diameters; the reflectivity difference is small around diameter of 200 nm, while, for small or large diameters, the reflectivity difference becomes larger. The reflection of the Au cap remains strong and is higher than 65% when the nanowire diameter is larger than 200 nm but decreases fast for diameters below 200 nm [20,30]. Thus, this is an additional advantage of the Au-catalyst growth method for GaAs nanowires. The reflectivity of the double gratings is much higher than that of the Au cap. However, the size of the Au cap is much smaller. When a lower threshold gain is needed, even the Au cap can be replaced by the double gratings. The adoption of double gratings enhances the end reflection and further reduces the threshold gain at a short nanowire length. The threshold of the laser is the lowest excitation level at which stimulated emission rather than spontaneous emission dominates inside the gain medium. The threshold gain $g_{th}$ is used to describe the required gain per unit length to achieve lasing and is defined as [31]:

(2) $$g_{th}=\frac{1}{\Gamma_{wg}}\left[\alpha_{i}+\frac{1}{L}\ln\left(\frac{1}{R}\right)\right]$$

where *L* is the length of the nanowire resonant cavity, and *R* denotes the geometric mean of the reflectivity of the two nanowire ends. $\Gamma_{wg}$, adopted here, is the modal confinement factor that indicates how well the waveguiding mode overlaps with the gain medium (i.e., GaAs nanowire) [32]. It is defined as the ratio between the modal gain and material gain within the active region [33,34]:

(3) $$\Gamma_{wg}=\frac{\frac{n_{a}}{2\eta_{0}}\int_{A_{a}}^{\;}d\rho {\left|E\left(\rho \right)\right|}^{2}}{\int_{A_{a}}^{\;}d\rho \frac{1}{2}Re\left[E\left(\rho \right)\times H^{*}\left(\rho \right)\right]\cdot \hat{z}}$$

where $\eta_{0}$ is the intrinsic impedance; $n_{a}$ is the refractive index of the active region; $A_{a}$ is the cross-section area of the active region; A is the whole cross-section area ideally extending to the infinity; and E and H are the complex electric and magnetic fields of the waveguiding modes. The threshold gain is one of key indicators of a laser and is crucial to the lasing. The threshold gains of the nanowire laser with double gratings, with single grating and without gratings for various nanowire diameters from 150 to 300 nm are depicted in Figure 7b. The nanowire length is 3 and 10 μm for comparison. All the threshold gains increase with the reducing nanowire diameter due to the weaker reflection and the modal confinement of the nanowire. For both nanowire lengths of 3 and 10 μm, the threshold gain of the nanowire with double gratings is the lowest, and the threshold gain of the nanowire without gratings is the highest, meaning that the double gratings benefit the lasing of the nanowire. For a nanowire diameter larger than 200 nm, the threshold gain of the nanowire with double gratings remains at a very low level of around 200 cm^−1^. Thus, lasing can be easily achieved in the nanowire without strong pump. Furthermore, the threshold gain of the nanowire with double gratings and a length of 3 μm achieves a similar level with that of the nanowire with no gratings and a length of 10 μm. It is necessary to note that the single grating reduces the threshold gain a lot compared to that without gratings, and the double gratings further reduce the threshold gain. However, the threshold gain difference between the single grating and double gratings is not large. From the above equation of threshold gain, the threshold gain is determined by both reflectivity and confinement factor. The reflectivity difference between double gratings and a single grating is not as large as that between double gratings and bare nanowire. So, as in the inset shown in Figure 7c, the end part with the Au cap is also sandwiched in the double Ag gratings, which is the same as the dielectric end facet to enhance the reflection. Consequently, the threshold gain of the nanowire with double gratings is dramatically reduced and much lower than that of the nanowire with single grating. Thus, the proposed short-period symmetric double gratings effectively reduce the threshold gain of the nanowire laser and make lasing emission easier under weak pump.

## 4. Conclusions

In summary, a novel structure of a sandwiched nanowire in double Ag gratings is proposed to provide high reflection for the nanowire. Due to the intense interaction between the gratings and the waveguiding mode within the nanowire, the double gratings have a short period of 5, making its length as short as 700 nm, together with high reflection. The reflectivity is higher when the length of the double gratings is assumed to be 1400 nm. The double gratings can achieve a high reflectivity of around 90% for various nanowire diameters from 150 to 300 nm. The reflection of the nanowire with a single grating was compared with that of double gratings. The reflectivity of the nanowire with double gratings is larger than that of the nanowire with a single grating for all nanowire diameters. The reflectivity difference is larger for larger or smaller nanowire diameters. For a short period of 5, the reflectivity of the nanowire with double gratings is much higher than that of the nanowire with a single grating. The threshold gain of the nanowire with double gratings and a length of 3 μm is similar to that of the nanowire with no gratings and a length of 10 μm. Moreover, by placing the end part with the Au cap inside the double gratings to form a symmetric structure, the threshold gain of the nanowire with double gratings is dramatically reduced. It is much lower than that of the nanowire with a single grating. Thus, the double gratings are effective in reducing both the threshold gain and the nanowire length. The proposed novel structure may have potential applications in on-chip photonic computing and interconnection.

## Data Availability

Not applicable.
